# Obesity and what we need do about it- an interview with John Wass

**DOI:** 10.1186/s12916-014-0135-0

**Published:** 2014-08-26

**Authors:** John Wass

**Affiliations:** Oxford Centre for Diabetes, Endocrinology and Metabolism, Churchill Hospital, Oxford, OX3 7LJ USA

**Keywords:** Education, Healthcare services, Obesity

## Abstract

In this podcast we talk to Professor John Wass, co-author of the ‘Action on obesity: Comprehensive care for all’ report, and Chair of the Working Party for Action on Obesity in the UK. In this interview Prof Wass discusses the gaps in care for obese patients in current UK healthcare services, and outlines his recommendations on what actions should be taken to tackle these issues, including how education about nutrition and obesity should be offered to the public as well as within the formal medical education system.

The podcast for this interview is available at: http://media.biomedcentral.com/content/movies/supplementary/johnwass-audio-v1.mp3.

## Introduction

**Figure 1:**
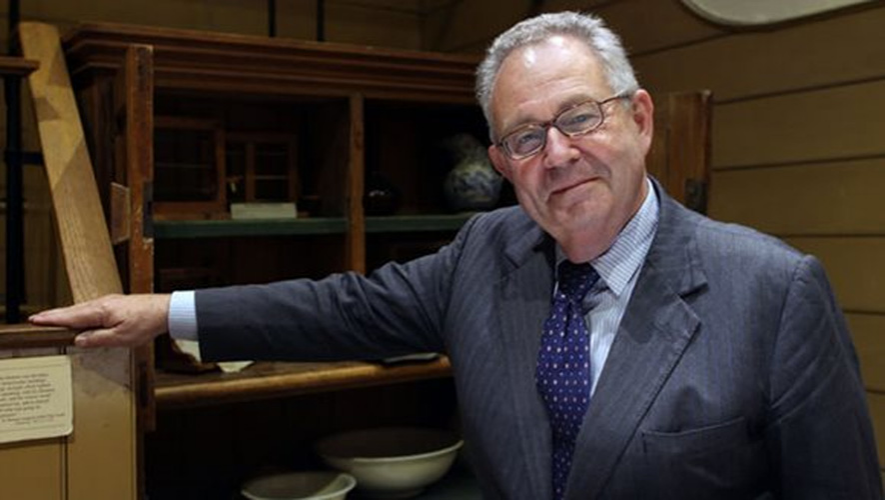
**John Wass.**

Prof John Wass (Figure 1) is a consultant physician based in Oxford, UK and is Professor of Endocrinology at Oxford University. Until 2011 he was also the Head of the Department of Endocrinology at the Oxford Centre for Diabetes, Endocrinology and Metabolism, Churchill Hospital Oxford, UK. He has been an elected council member of the Royal College of Physicians since 2006.

In January 2013, Prof Wass co-authored the ‘**Action on Obesity’** report for the Royal College of Physicians in which, together with the working party he chaired, noted that nearly a quarter of the UK population is obese. In this interview, we talk to Prof Wass about some of the key goals of the working group which aims to actively curb the rise of obesity by filling current gaps in healthcare services for obese patients and healthcare workers, and to improve awareness about nutrition and obesity within the public as well as the medical education system.

The edited podcast for this interview is available at: http://media.biomedcentral.com/content/movies/supplementary/johnwass-audio-v1.mp3.

## Edited transcript

### 1. You are an expert in the field of endocrinology, and in recent years have become increasing involved in recommending what actions can be taken to tackle the prevalence of obesity. Can you explain to us why you’re interested in obesity in particular?

As an endocrinologist, I’ve been taking an increasing interest in the management of obesity. Endocrine and diabetes physicians tend to look after patients who are obese and there are huge gaps in the treatment of patients who are obese up and down the country. So, when I was asked to do a report for the (Royal) College of Physicians, I was very keen. That report was published last year, and recommended that we put in place, as far as we could, improved services for people who have an obesity problem. That’s why I, as an endocrinologist, am doing it and being so - hopefully, proactive.

### 2. You’re certainly being proactive - you’re currently Academic Vice President of the Royal College of Physicians, and also the Chair of the Working Party for Action on Obesity. You mentioned the RCP report that you co-authored last year. In that, you’ve outlined some key actions that can be taken to tackle the rise of obesity within the UK specifically. Can you briefly take us through the goals of the working group and the findings of the report?

First, we did a survey and found huge gaps in the care of patients with an obesity problem. Having done that, we aim to try and fill them within the next six to 12 months.

The second thing is that we wanted to get a together a group of physicians who are interested in bariatric medicine. We’ve done that and they’re now a cohort in the Society of Endocrinology.

The third thing we wanted to do was to get a patient charter going, and it should be published within the next couple of months.

The next thing we wanted to do was to try and improve the health of healthcare workers who have a higher prevalence of obesity, and we’re doing some work with a number of others, including Carol Black and Sian Williams at the College of Physicians and others in the Department of Health, to try and improve that in the hospitals where people are being looked after and who are healthcare workers themselves.

So those are some of the key actions and some of the key results of the work over the last six months or so.

### 3. Do you think these kinds of actions that you’re taking in the UK can be generalised across other developed countries?

I would hope so. I haven’t actually studied the delineation of services in other developed countries, and whether there are similar gaps to those that there are in the UK, to be honest with you. I know, of course, that our country has a high prevalence of obesity so it is something which is very important. But I haven’t looked at other countries. I’d like to get on, at some stage, to the various tiers of service that there are because those are important. We’re also now developing those in a logical and coordinated way.

### 4. As far as I understand, the rate of increase of obesity in the UK is second only to the US. Is that correct?

Yes. There are some of the Middle Eastern countries that similarly have a problem actually, but yes that’s correct.

### 5. So what role does education play in this? Is trying to inform the public about the negative impact of obesity on health enough, or is there more that can be done within the health services sector specifically?

I think, specifically, there are a lot of things that can be done. I think that in Tier 1, which is the responsibility of Public Health England, there is a huge opportunity to educate the public and I’m very much protagonistic to the idea of public-private sector collaboration. For example, some supermarkets are now trying to encourage public education of healthy eating and so on. The role of education is absolutely key.

One of the things we’re doing, which I haven’t mentioned before, is to make sure that all the medical disciplines have education on nutrition and obesity, and also that the core medical trainees have education on the same topics. We’re also doing some work trying to make sure that all the medical schools have this because it is not something which they’re currently exposed to in any great depth. So there are key issues of education. That’s the medical and quasi-medical parts of it but, in fact, also patients and the public need to be educated as much as possible.

### 6. Regarding any kind of intervention – there’s normally a significant cost associated. Have there been any studies looking at the cost-effectiveness of obesity intervention programs within the UK?

There have been cost-effectiveness investigations in Australia, most particularly, and indeed in the UK as well. Cost-effective interventions (reviewed in *The Lancet* in August 2011) include– gastric banding. Gastric operations are very cost-effective, they reduce the cost of anti-diabetic drugs, anti-hypertensive drugs, and so gastric surgery is cost-effective. There are also assessments of the effectiveness of programmes such as Weight Watchers. So there are some private sector organisations that can very effectively, and cost-effectively, help to reduce weight.

### 7. Is there anything else that can be done to further decrease the obesity levels? In particular, I’m interested to know your opinion about what role the food industry or the government can take in tackling this.

Public Health England is key in educating people. I think that the food industry has got to, as far possible, work with everybody else to try and improve some of the ways foods are sold. Some are already behaving responsibly in the food and responsibility deals, which are run by the Department of Health. I think more could be done in this area. But there are huge difficulties, I would say, between commercialisation and how companies wish to sell things - and actually the combativeness, if you like, of this, versus the health of the nation. I think it’s a very difficult path to tread between pushing the sale of foods which may have a lot of sugar in, and actually increasing the levels of obesity.

### 8. Finally, in your opinion, is obesity a disease?

I think it is a disease, actually. I think it needs to be actively managed, it’s something for which there are treatments, and by all other counts I think it’s a disease which we need to take a lot more seriously in this country. That goes for government legislating for various reasons, and from healthcare individuals to the general population – every person in the country to take it more seriously and to lead more healthy lives. I think there’s a lot of education, a lot of examples and so on, that need to be more actively promulgated. At the moment, when people go to their general practitioners, or when they come into hospital, it’s not necessarily noted if they are obese. This should be something which is actually marked up as a diagnosis so that it’s taken seriously. I think that will help individuals address the problem that they have.

### 9. Where can I find out more?

See reference list [1,2].
